# Pheromone-Induced Odor Associative Fear Learning in Rats

**DOI:** 10.1038/s41598-018-36023-w

**Published:** 2018-12-07

**Authors:** Samantha J. Carew, Bandhan Mukherjee, Iain T. K. MacIntyre, Abhinaba Ghosh, Sa Li, Gilbert J. Kirouac, Carolyn W. Harley, Qi Yuan

**Affiliations:** 1 Biomedical Sciences, Faculty of Medicine, Memorial University of Newfoundland, St. John’s, A1B 3V6 Canada; 2 Department of Oral Biology and Psychiatry, Rady Faculty of Health Sciences, University of Manitoba, Winnipeg, R3E 0W2 Canada; 3 Psychology Department, Faculty of Science, Memorial University of Newfoundland, St. John’s, A1B 3X9 Canada

## Abstract

Alarm pheromones alert conspecifics to the presence of danger. Can pheromone communication aid in learning specific cues? Such facilitation has an evident evolutionary advantage. We use two associative learning paradigms to test this hypothesis. The first is stressed cage mate-induced conditioning. One pair-housed adult rat received 4 pairings of terpinene + shock over 30 min. Ten minutes after return to the home cage, its companion rat was removed and exposed to terpinene. Single-housed controls were exposed to either terpinene or shock only. Companion rats showed terpinene-specific freezing, which was prevented by β-adrenoceptor blockade. Using *Arc* to index neuronal activation in response to terpinene re-exposure, stressed cage-mate induced associative learning was measured. Companion rats showed increased neuronal activity in the accessory olfactory bulb, while terpinene + shock-conditioned rats showed increased activity in the main olfactory bulb. Both groups had enhanced activity in the anterior basolateral amygdala and central amygdala. To test involvement of pheromone mediation, in the 2nd paradigm, we paired terpinene with soiled bedding from odor + shock rats or a rat alarm pheromone. Both conditioning increased rats’ freezing to terpinene. Blocking NMDA receptors in the basolateral amygdala prevented odor-specific learning suggesting shock and pheromone-paired pathways converge in the amygdala. An alarm pheromone thus enables cue-specific learning as well as signalling danger.

## Introduction

Fear learning is evolutionarily essential for species’ survival, without which dangerous situations may lead to not only the death of individuals but eventual extinction of the species. Fortunately, one does not have to directly experience danger in order to acquire an adaptive fear; there are different means for the communication of fear. Humans explore their environment crucially through vision and communicate extensively through languages. Humans can learn fear through the observations of others’ experiences. Similarly, just hearing a frightening recollection can lead to avoidance of a dangerous situation in the future. Other animals, such as rodents, rely heavily on their sense of smell to navigate their environment and to communicate. An alarm pheromone is a type of volatile pheromone that is released by rodents when they are stressed^1–4^. These molecules, even in minute amounts, can be detected by the vomeronasal system and main olfactory system of another member of the same species (conspecific) and evoke anxiety or fear reactions in the conspecifics^4–6^. A rat alarm pheromone released in response to perianal stimulation and footshock has recently been identified^2^. The release of this pheromone is associated with increases in anxiety behaviors^7^, hyperthermia to stress events^8^, and increased auditory startle responses^9^ in receiving rats. Thus, alarm pheromones can prepare conspecifics for potential danger and thereby aid in the communication of fear to conspecifics without their direct exposure to an inherently stressful situation. But is cue specific fear learning engaged by an alarm pheromone?

The nature of fear learning is essentially associative. A sight, a sound or a smell associated with a harmful stimulus, can later trigger the recall of the fearful event. This is commonly modeled in rodents by classical conditioning, in which a harmful stimulus (unconditioned stimulus, UCS) is presented concurrently with a neutral stimulus (conditioned stimulus, CS). Animals develop fearful or defensive responses to the neutral stimulus. Can such classically conditioned fear be transferred to another conspecific in the absence of an external aversive stimulus through pheromone communication? If so, does pheromone-mediated memory utilize distinct neural circuitry from that which mediates classically conditioned fear?

We developed a pheromone fear communication model in adult rats by housing naïve non-stressed rats (companions) with odor/shock-conditioned rats. The companion rats developed conditioned freezing to an odor that they were exposed to shortly after housing with the odor/shock-conditioned rats. Replacing the odor/shock conditioned rats with their soiled bedding or with the alarm pheromone molecules 4-methylpentanal plus hexanal^2^ produced similar odor fear conditioning in naïve odor-exposed rats. To determine whether these two differentially acquired memories (classical odor conditioning *vs* pheromone odor conditioning) utilize distinct neural circuitries, immediate early genes *Arc* and *Homer1a (H1a)* were used to map cells activated by the conditioned odor and a novel control odor across several regions of the brain. We included brain structures specialized for specific odor processing such as the main olfactory bulb (MOB), olfactory tubercle (OT), and piriform cortex (PC)^10,11^; for pheromone processing such as accessory olfactory bulb (AOB), and the medial amygdala (MeA)^10–12^; and for fear memory formation such as lateral amygdala (LA), basolateral amygdala (BLA) and central amygdala (CeA)^13–16^. Our results reveal converging pathways in the common fear circuitry of the amygdala for both types of learning.

## Materials and Methods

### Subjects

Sprague Dawley rats (8–10 weeks old, weight 200–300 g, in good health) of both sexes (*n* = 110 total) were assigned randomly to groups. Rats were housed in polycarbonate cages on a 12 h light/dark cycle, given *ad libitum* access to food and water, with all behavioural manipulation completed during the light phase of the light cycle. Odor + shock conditioned rats (O^+^*/*S^+^) were housed with same sex companion rats one week before the experiments and for the duration of the experiments, while other groups were housed alone. All procedures were approved by the Memorial University Institutional Animal Care Committee and carried out in compliance with the guidelines of the Canadian Council on Animal Care.

### Experimental designs and statistical analysis

#### Experiment 1: Stressed cage mate-induced odor associative learning

Behavioral study: Odorants: Odorants were diluted with mineral oil to specific concentrations. Odorants used were terpinene (6.63%) and octanol (2.67%). These odorants were chosen as they are neither innately appetitive nor aversive to adult rats, and the concentrations were chosen so that the odors would emit a vapor-phase partial pressure of 1 Pascal^17^.

Apparatus: All behavioural training and testing was completed with a custom-made olfactometer for air and odorant delivery attached to the shock chamber: a plexiglass chamber that sits on top of an electrified grid, connected to a shock generator/scrambler (Muromachi Kikai Model SGS-003DX). Polyvinyl carbonate bottles were used for each odor and connected to the olfactometer by C-flex tubing pinched shut when not in use. Evacuation tubing with a fan was attached to the top lid of the shock chamber to promote odor removal.

Odor conditioning and testing: All rats were habituated to the shock chamber for one 30 min session each on two consecutive days with clean air pumped through the shock chamber. On the third day, rats were trained individually with four separate exposures to either odor, shock, or odor and shock, depending on their respective groups at 5, 15, 20, and 30 min during a 30 min training session. Odorant (terpinene) was delivered for 1 min at each time point. Shock was delivered at the last sec of the odor delivery (0.5 mA for 1 sec). Between each experiment, the shock chamber and grids were thoroughly cleaned with 70% ethanol and clean paper towels. There was a 15 min interval between the chamber cleaning and next experiment, where residual smell and ethanol were removed *via* the evacuation tubing. The fourth day consisted of a 30 min behavioural testing session in the same conditioning chamber. Medical air was delivered in the first half of the session and an odorant was delivered in the second half. Rats were tested with terpinene and octanol (a control odor) on the same day, and the order of the odorant testing was randomized and counter-balanced. The percentage of freezing time in response to the terpinene and octanol exposure was measured.

Six groups were examined: (1) O^−^/S^+^, rats were housed alone and received shock only, no odor during the experiment; (2) O^+^/S^−^, rats were housed alone and received terpinene odor but no shock during the training; (3) O^+^/S^+^, rats received both terpinene odor and shock; and (4) O^+^/Comp (companion), rats were housed with O^+^/S^+^ rats and exposed to terpinene only during the training. O^+^/S^+^ rats were returned to the cages with O^+^/Comp rats immediately following the odor/shock conditioning. Ten minutes later, O^+^/Comp rats were subjected to the odor only conditioning. A subset of the O^+^/Comp rats in this group received saline (50 µl, i.p.) during the habituation and 40 min before the training; (5) O^+^/Comp + Prop, O^+^/Comp rats received saline during the habituation and propranolol (20 mg/kg, i.p.) 40 min before the training. (6) O^−^/Comp, companion rats were housed with the O^+^/S^+^ rats but not exposed to the conditioned odor.

Neural circuit mapping: A separate cohort trained identically to groups 1–4 as described above underwent tissue collection for *Arc* and *H1a* mRNA visualization on the 4^th^ day. Animals were given a final odor exposure in lieu of behavioural testing. Rats were placed in a sealed plexiglass container ventilated with a continuous flow of charcoal-filtered air for 1.5 hrs. Rats were then given a 5 min exposure to octanol, another 20 min of charcoal-filtered air, then a 5 min exposure to the conditioned odor, terpinene, followed by immediate isoflurane anaesthesia and decapitation. Brains were collected and flash frozen in 2-methylbutane immersed in an ethanol/dry ice slurry and kept at −80 °C.

Fluorescence *in situ* hybridization (FISH): Brains were trimmed so that the cerebellum was discarded, and only the right hemisphere was analyzed. The right hemispheres of rats from each behavioural group were arranged so that the rostral end of their olfactory bulbs touched a razor blade to align them at the same rostral-caudal level. Brains were then arranged in a custom-made plastic box filled with OCT medium at −20 °C in a cryostat and frozen in a block. Coronal sections of 20 μm were collected on 2% 3-aminopropyltriethoxysilane treated slides. Five representative slides over the rostral-caudal range of each of the MOB, AOB, aPC/tubercle, and pPC/amygdala were chosen for FISH and stored at −20 °C.

The double FISH protocol was established previously^18^. Briefly, full length *Arc* riboprobes conjugated to digoxigenin and *H1a* riboprobes conjugated to fluorescein were obtained using commercial transcription kits (Maxiscript) and RNA labeling mixes (Roche). Riboprobes were purified using RNA mini quickspin columns (Roche) and verified via agarose gel.

Slides were thawed for 30 minutes at room temperature, fixed in 4% paraformaldehyde at 4 °C, bathed in acetic anhydride and acetone/methanol (Fisher Scientific), and treated with pre-hybridization buffer and hybridization buffer (Sigma-Aldrich) containing *Arc* and *H1a* probes. Slides were hybridized overnight in a 56 °C oven. All steps until this point were performed in the absence of RNAse. Slides were washed in a series of sodium citrate solutions followed by cleavage of any remaining single-stranded RNA using RNAse A. Endogenous peroxidases were quenched with H_2_O_2_ and slides blocked with 5% sheep serum (Sigma-Aldrich). *Arc* riboprobe was detected with anti-digoxigenin-POD (Roche) and a TSA cyanine-3 substrate kit (Perkin Elmer). Following *Arc* detection slides were dipped in 2% H_2_O_2_ solution to quench any residual HRP activity. *H1a* riboprobe was detected with anti-fluorescein-POD (Roche) and a TSA Fluorescein Tyramide substrate kit (Perkin Elmer). Nuclei were counterstained with DAPI (Sigma-Aldrich). Slides were coverslipped with Vectashield antifade medium (Vector Laboratories), sealed with clear nail polish, and kept at 4 °C before confocal microscopy scanning.

Image Acquisition and Analysis: All slides were scanned in a Fluoview FV1000 confocal microscope (Olympus). All images were taken at 20X magnification. The photomultiplier tube assignments, confocal aperture size, and contrast remained constant for each slide. The z-stacks (optical thickness: 1.0 μm) were taken throughout the thickness of the section and were acquired from 3–4 slides for each animal.

The mitral cell layer was analyzed in the olfactory bulbs, including the dorsolateral and ventral medial regions in the MOB. Layer II was analyzed in the PC, and the dense cell layer was analyzed in the OT. Images were analyzed from the center of each of the amygdala subdivisions.

ImageJ software was used for counting cells in the scanned images. In all areas except the OBs total cell counting was done automatically for the DAPI stained nuclei; images were cropped to include only the area of analysis, transformed to binary images (black and white), and cells were counted using the “Analyze Particles” function in ImageJ. For the *H1a*^+^ and *Arc*^+^ cells, counting was done manually by checking 20% of the mid-range of the stack that comprised each cell. Average cell counts of *Arc*^+^ cells were divided by the average cell counts of *H1a*^+^ cells to compute a ratio of cells active to the conditioned odor versus cells active to the control odor for each animal.

#### Experiment 2: Pheromone-induced odor associative learning

Behavioral study: Four groups were included: (1) O^+^/S^−^ (terpinene odor only); (2) Ph-T (pheromone paired with terpinene). Rats were housed alone and exposed to the clean bedding with a piece of filter paper soaked with 0.75 mL 4-methylpentanal (1.3 × 10^−6^ M) and hexanal (8.7 × 10^−6^ M) binary mixture (dissolved in purified water)^2^ on top of the bedding and received terpinene as the conditioned odor. (3) SB-T (soiled-bedding conditioned with terpinene); (4) SB-Oc (soiled-bedding conditioned with octanol). Rats were housed alone and exposed to the soiled bedding. A donor rat was shocked to release pheromone in the shock chamber (4 shocks during 30 min). The soiled bedding was woodchip bedding placed underneath the shock chamber during the donor rat shock and was subsequently left untouched for the conditioning of the SB rat.

Habituation, odor delivery during the training, and testing were carried out in the same manner as in Experiment 1, except testing lasted 10 min (5 min in clean air, 5 min in an odorant), instead of 30 min. Additionally, Experiment 1 and 2 were carried out in two different rooms with different experimenters.

To study the role of NMDA receptors in the basolateral amygdala, a separate cohort underwent cannular implantations. Cannular surgeries were performed 1 week before the behavioral experiments. During surgeries, rats were anesthetized with isoflurane gas and secured in a stereotaxic apparatus. Two holes were drilled 2.5 mm posterior, and 4.9 mm bilateral relative to bregma for the BLA. Guide cannulae were inserted 7.8 mm ventral to the skull surface. Guide cannulae were secured by dental cement to two skull screws. The skin was sutured and the rats were returned to their cages for recovery.

O^+^/S^+^ and pheromone molecule conditioned (O^+^/Ph) rats were infused with either saline or D-APV (5 mM; 1 µl) bilaterally into the BLAs 30 min before the conditioning experiments. Infusion tubing and cannular attachment were performed during habituation for animals to become acclimated to the attachment of the infusion tubing.

### Statistics

**Figure 1:**
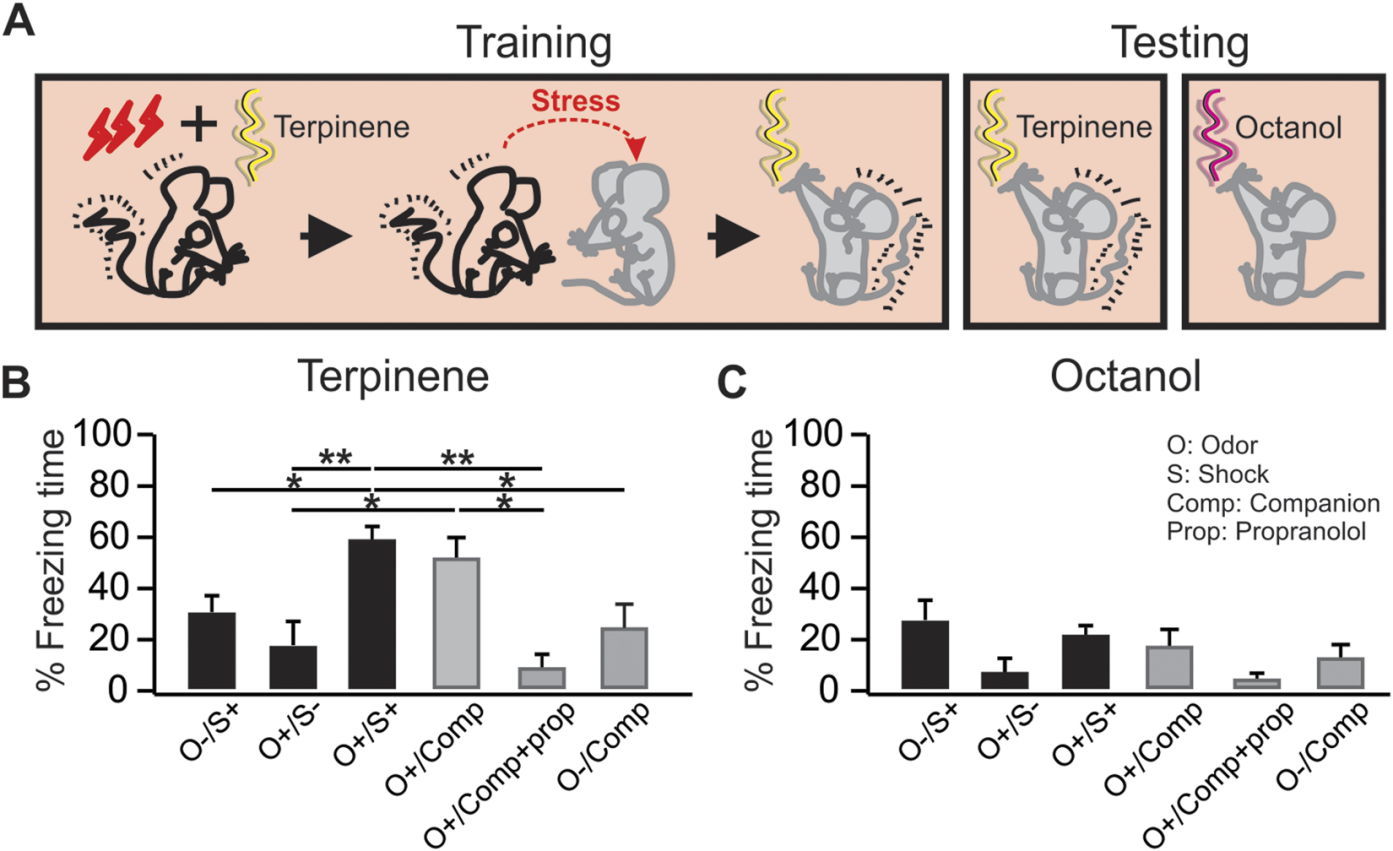
Conditioned fear can be transmitted to conspecifics in the absence of an external aversive stimulus. (**A**) Schematics of the odor conditioning and testing paradigm. (**B**) Percentage freezing time during the testing to the conditioned odor terpinene. (**C**) Percentage freezing time during the testing to the novel control odor octanol. O^−^/S^+^, shock only rats; O^+^/S^−^, odor only rats that were caged alone; O^+^/S^+^, odor/shock conditioned rats; O^+^/Comp, odor only rats that were caged with odor/shock conditioned rats; O^+^/Comp + Prop: O^+^/S^−^ comp rats that were injected propranolol before training; O^−^/Comp: companion rats without subsequent odor exposure. *p < 0.05, **p < 0.01. Error bars, mean ± SEM.

**Figure 2:**
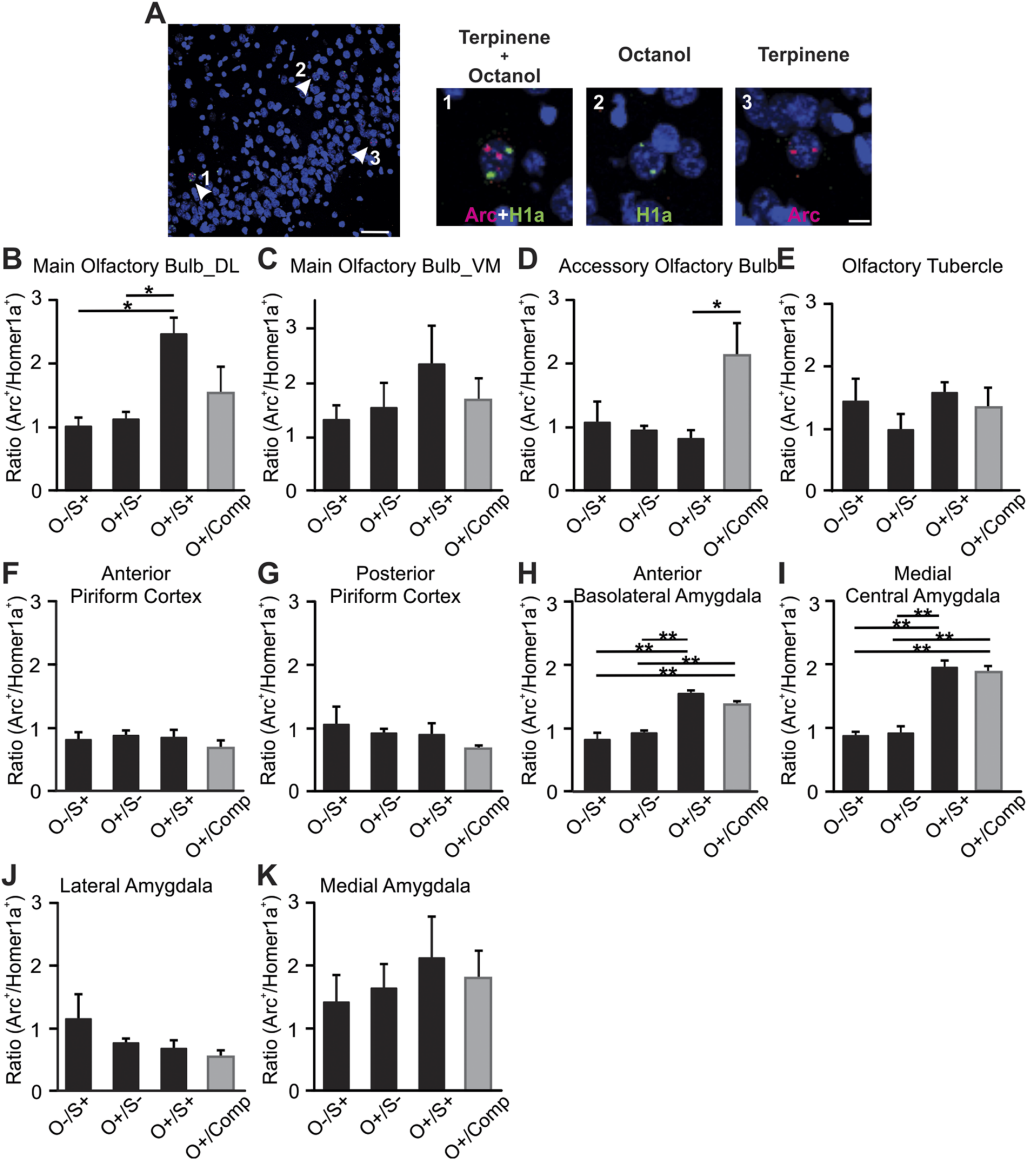
Pheromone odor conditioning and classical odor conditioning activate distinct but converging circuitries in the brain. (**A**) An example of *Arc* and *H1a* mRNA staining. “1” indicates a double labeled cell (green and red in the nucleus) that was activated by both terpinene and octanol; “2” indicates a cell expressing *H1A* (green; activated by octanol); “3” indicates a cell expressing *Arc* (red; activated by terpinene). Scale bars, 100 and 20 µm. (**B**–**K**) Ratios of *Arc*^+^/*H1A*^+^ cells in various olfactory and limbic structures. O^−^/S^+^, shock only rats; O^+^/S^−^, odor only rats that were caged alone; O^+^/S^+^, odor/shock conditioned rats; O^+^/Comp, odor only rats that were caged with odor/shock conditioned rats. *p < 0.05, **p < 0.01. Error bars, mean ± SEM.

**Figure 3:**
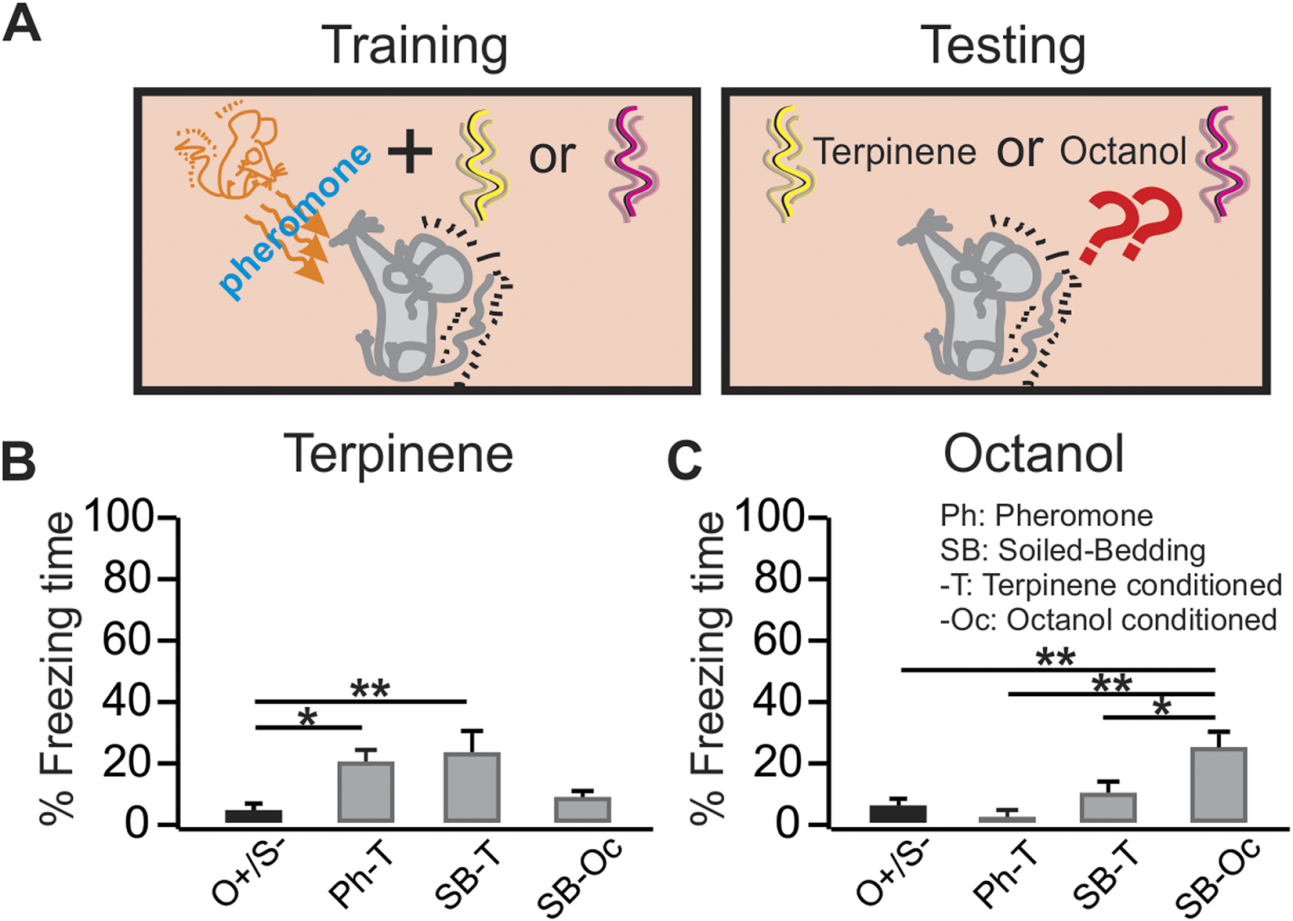
Alarm pheromone mediates the fear learning in companion rats. (**A**) Schematics of the odor conditioning and testing paradigm. (**B**) Percentage freezing time during the testing to the odor terpinene. (**C**) Percentage freezing time during the testing to the odor octanol. O^+^/S^−^, odor only rats that were caged alone; Ph-T, terpinene odor exposed rats that were conditioned with previously identified alarm pheromone molecules; SB-T, terpinene exposed rats that were conditioned with soiled bedding; SB-O, octanol exposed rats that were conditioned with soiled bedding. *p < 0.05, **p < 0.01. Error bars, mean ± SEM.

**Figure 4:**
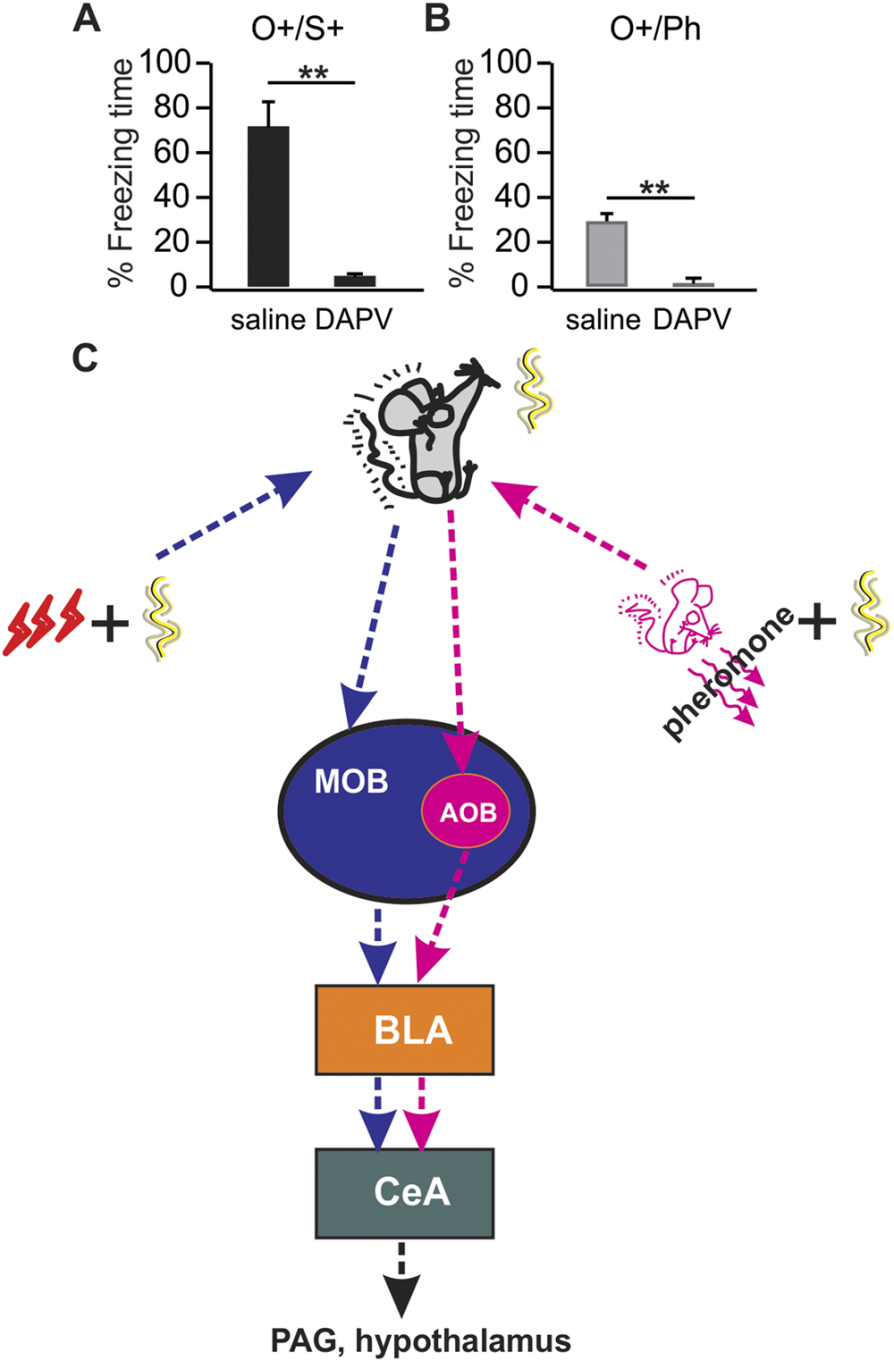
D-APV bilateral BLA infusions prevents both pheromone odor conditioning and classical odor conditioning. (**A**) Percentage freezing time during the testing to the conditioned odor terpinene in odor/shock conditioned (O^+^/S^+^) rats, infused with either D-APV or saline. (**B**) Percentage freezing time during the testing to the conditioned odor terpinene in pheromone molecule conditioned rats (O^+^/Ph), infused with either D-APV or saline. **p < 0.01. Error bars, mean ± SEM. (**C**) Converging pathways of classical and pheromone fear conditioning in rats. MOB: main olfactory bulb; AOB: accessory olfactory bulb; BLA: basolateral amygdala; CeA: central amygdala; PAG: periaqueductal grey.

OriginPro 9.0 was used to analyze the datasets. One-way ANOVAs plus *post-hoc* Bonferroni tests were used to compare different groups in Figs 1–3. A two sample t-test (2-tail) was used in Fig. 4. Data are presented as mean ± SEM in Results and Figures.

## Results

### Conditioned fear can be transmitted to conspecifics in the absence of an aversive stimulus

In the first experiment, we tested whether the companion rats (O^+^/Comp) of the O^+^/S^+^ conditioned rats were able to form cue-specific fear memory when subsequently exposed to the conditioned odor terpinene (Fig. 1A). If so, does the stress transferred from the O^+^/S^+^ to the O^+^/Comp rats serve as the UCS in the conditioning of the O^+^/Comp rats? To test this, a subset of the companion were injected with an anxiolytic β-adrenoceptor antagonist propranolol (O^+^/Comp + Prop) before the training. Additionally, we tested where the CS-UCS association occurs by including a group of companion rats that were not subsequently exposed to the conditioned odor (O^−^/Comp). The absence of learning in the O^−^/Comp group would suggest that any residual terpinene smell on the O^+^/S^+^ rat is not sufficient to induce associative learning in the O^+^/Comp rat during the social interaction, although it does not exclude any priming effects of social interaction on subsequent odor conditioning.

Like the O^+^/S^+^ rats, the O^+^/Comp rats developed significant freezing to terpinene. There was a significant difference in the percentage of freezing among different groups in the presence of terpinene (F_5,46_ = 8.16, p = 1.41E-5, ANOVA; Fig. 1B). *Post-hoc* Bonferroni tests showed significant differences between the O^+^/S^+^ (58.91 ± 4.55%, n = 19) and O^+^/S^−^ (17.69 ± 8.92%; n = 6, t = 4.34, p = 0.001), and between the O^+^/S^−^ and O^+^/Comp (54.94 ± 9.08%; n = 10, t = 3.27, p = 0.031). Pre-training infusion of propranolol (O^+^/Comp + Prop) prevented the formation of odor-specific memory (9.5 ± 3.84%; n = 4, t = 3.53, p = 0.014, compared to the O^+^/Comp rats). The companion rats without terpinene exposure (O^−^/Comp) spent significantly less time freezing in terpinene (25.39 ± 7.46, n = 7) than the O^+^/S^+^ rats (t = 3.53, p = 0.015). No significant difference was observed in animals exposed to the control odor octanol (F_5,44_ = 1.67, p = 0.16; Fig. 1C), or in their baseline freezing level before the odor exposure during testing (Supplementary Fig. 1A,B). Further analysis separating sex groups revealed no differences in female and male performance in either the O^+^/S^+^ or the O^+^/Comp groups (Supplementary Fig. 2). These results suggest the learning in the companion rat is dependent on norepinephrine (NE) release, likely induced by the transfer of the stress from the O^+^/S^+^ rats. Exposure to the conditioned odor following the interaction with a stressed rat was necessary for specific fear odor memory formation in the companion rat. Additionally, we show that the odor-specific learning is not contingent on the training context, as conditioned rats tested in a different context also showed significant freezing to the conditioned odor (Supplementary Fig. 3) and no general anxiety in an elevated plus maze test (Supplementary Fig. 4).

### Stressed cage mate-induced odor conditioning activates a classical amygdala fear pathway

We next measured activation profiles of several brain regions critically involved in odor or pheromone processing and fear memory formation. We employed cellular compartment analysis of temporal activity by fluorescence *in situ* hybridization (catFISH)^19^. This technique utilizes the immediate-early genes *H1A* and *Arc* to visualize cells that are active to two temporally distinct events. *H1A* is expressed in the nucleus approximately thirty minutes following a stimulus, while *Arc* appears in the nucleus approximately five minutes after stimulus presentation^18^. Four groups were used for this experiment: O^−^/S^+^, O^+^/S^−^, O^+^/S^+^ and O^+^/Comp. Twenty-four hours following odor conditioning, rats were exposed to octanol for 5 min, clean air for 20 min, terpinene for 5 min, and then immediately sacrificed. Cells expressing *H1A* were those activated by the control odor octanol, while cells expressing *Arc* were those activated by the conditioned odor terpinene (Fig. 2A). We systematically measured the *H1A* and *Arc* expression in the MOB (Fig. 2B,C), AOB (Fig. 2D), OT (Fig. 2E), sub-regions of the PC (Fig. 2F,G), and several nuclei of the amygdala (Fig. 2H–K).

We measured ratios of *Arc/H1A* (the ratio of the number of terpinene-activated cells over that activated by octanol) as a way of normalizing the activation profiles in each region. This within-tissue control protocol reduces variation from intrinsic variability in individual animal response levels to odor input and from variability related to differences in tissue processing. This approach enhanced the signal to noise ratio and resulted in the use of fewer animals for statistical comparisons (compare Fig. 2 with Supplementary Fig. 5, in which the activations of *Arc* and *H1A* were reported individually). Significant differences among groups were observed in the dorsal lateral MOB (F_3,12_ = 4.89, p = 0.02; Fig. 2B), AOB (F_3,12_ = 4.30, p = 0.03; Fig. 2D), anterior BLA (F_3,12_ = 23.05, p = 2.87E-5; Fig. 2H) and medial portion of the CeA (F_3,12_ = 49.65, p = 4.87E-7; Fig. 2I). O^+^/S^+^ rats showed significantly more activation in the dorsal lateral MOB (2.35 ± 0.37, n = 4) than O^−^/S^+^ (0.95 ± 0.15; n = 4, t = 3.45, p = 0.022), and O^+^/S^−^ rats (1.07 ± 0.07, n = 4, t = 3.15, p = 0.037; Fig. 2B). O^+^/S^+^ rats also showed enhanced activation in the anterior BLA (1.54 ± 0.09, n = 4) compared to O^−^/S^+^ (0.84 ± 0.9, n = 4, t = 7.04, p = 8.12E-5), and O^+^/S^−^ rats (0.93 ± 0.03, n = 4, t = 6.15, n = 4, p = 2.99E-4; Fig. 2H); and in the CeA (1.96 ± 0.10, n = 4) compared to O^−^/S^+^ (0.88 ± 0.07, n = 4, t = 9.13, p = 5.71E-6), and O^+^/S^−^ rats (0.92 ± 0.08, t = 8.77, p = 8.70E-6; Fig. 2I). In contrast, O^+^/Comp rats showed more activation in the AOB (2.14 ± 0.47, n = 4), significantly different from O^+^/S^+^ (1.06 ± 0.33, n = 4, t = 3.20, p = 0.045; Fig. 2D). Interestingly however, the O^+^/Comp rats also showed enhanced activation in the anterior BLA (1.37 ± 0.06, n = 4) compared to O^−^/S^+^ (t = 5.28, p = 0.001), and O^+^/S^−^ rats (t = 4.38, p = 0.005; Fig. 2H); and in the CeA (1.88 ± 0.08, n = 4), compared to O^−^/S^+^ (t = 8.46, p = 1.27E-5), and O^+^/S^−^ rats (t = 8.10, p = 1.99E-5; Fig. 2I). These results suggest that MOB and AOB hold the initial classically conditioned odor and socially transferred stress conditioned odor memory traces respectively, and then both conditioning pathways converge on the amygdala fear circuitry to generate conditioned freezing behavior upon re-exposure to the conditioned odor.

### Pheromone mediates the conditioned fear in conspecifics

Rats emit a series of ultrasonic calls when confronting distressful stimuli^20^. To determine whether rats transmit a fear state that supports conditioning by ultrasonic or alarm pheromone communication, we performed additional experiments. One group of rats were exposed to the previously identified shock-induced alarm pheromone molecules 4-methylpentanal and hexanal^2^ during the terpinene exposure (Ph-T). Another group of rats were exposed to the soiled bedding (SB) from the donor shocked rats, but were never physically in contact with the donor rats. A subgroup of the SB rats were conditioned with terpinene (SB-T) while another subgroup was conditioned with octanol (SB-Oc). All rats were tested for freezing in the presence of terpinene and octanol separately (Fig. 3A). There was a significant treatment effect to the terpinene (F_3,21_ = 6.37, p = 0.003; Fig. 3B) and octanol (F_3,21_ = 9.04, p = 4.8E-4; Fig. 3C). Consistent with pheromone-mediation of odor-specific conditioning, when trained with terpinene as the conditioned odor, the Ph-T group (20.58 ± 5.39, n = 4) showed significantly more freezing to terpinene than the control O^+^/S^−^ group (4.58 ± 1.93, n = 9, t = 2.89, p = 0.041). The SB-T group also showed more freezing (23.65 ± 6.67, n = 6) compared to the O^+^/S^−^ group (t = 3.94, p = 0.004; Fig. 3B). In contrast, when SB rats were tested with octanol, the SB-Oc group showed significantly more freezing (25.32 ± 3.86; n = 6) than the SB-T group (9.87 ± 4.20; n = 6, t = 3.32, p = 0.020), or the O^+^/S^−^ group (6.45 ± 2.26, n = 9, t = 4.44, p = 0.001; Fig. 3C). These experiments establish that rats can communicate fear and induce specific odor fear learning *via* pheromone information.

### Basolateral amygdala serves as the common plasticity locus for classical and pheromone conditioning

The BLA has been selectively implicated in valence learning, including the encoding of odor cue valence^21,22^. The anterior BLA contains a subpopulation of neurons that respond specifically to aversive stimuli such as shock^23^, consistent with our analysis showing enhanced activation to the shock conditioned odor in anterior BLA. We infused the NMDA receptor antagonist D-APV bilaterally into the BLA during either classical (O^+^/S^+^) or pheromone conditioned training (using alarm pheromone molecules 4-methylpentanal and hexanal as the UCS, O^+^/Ph) and tested freezing behavior 24 hr later. Both forms of learning were prevented by the D-APV infusions. In O^+^/S^+^ rats, the D-APV infused group (4.17 ± 1.77, n = 4) showed significantly less freezing than the saline infused control group (72.7 ± 10.20; n = 3, t = 7.79, p = 5.57E-4; Fig. 4A). In O^+^/Ph rats, the D-APV infused group (1.75 ± 1.42, n = 4) also spent significantly less time freezing than the saline infused group (29 ± 3.51; n = 3, t = 8.05, p = 4.80E-4; Fig. 4B). This establishes that the BLA is a common plasticity site for both classical odor conditioning and pheromone learning.

To further illuminate the routes of information processing from the upstream structures, we injected the retrograde tracer cholera toxin subunit B conjugated to Alexa Fluor-488 unilaterally into the BLA. One week later, we observed robust labeling of neurons in the PC, MeA and CoA, with sparser labeling in the MOB and AOB (Supplementary Fig. 6). Thus, odor and pheromone information could directly transmit to the BLA from the MOB and the AOB, or *via* the MeA, CoA or PC^10–12^.

## Discussion

When stressed, rodents release a type of pheromone which provokes anxiety or fearful responses in conspecifics^2,4,5^ or even in themselves^4,24^. This type of pheromone, termed alarm pheromone, increases anxiety and can lead to conspecific avoidance of the immediate danger through increased defensive and risk assessment behaviour^7^, thus promoting survival of the species at the price of only a few animals experiencing the real danger. However, if the effect of alarm pheromone is short-lived, conspecifics that are preserved through an alarm pheromone communication could be compromised when confronting the same danger in the future. To be evolutionarily advantageous, animals should be able to learn to associate relevant cues with alarm and obtain the advantage of avoiding the danger in the future by recognizing those cues. Can a memory trace be formed through alarm pheromone and cue association? Our research suggests this is the case. Either being caged with stressed rats, being placed over soiled bedding from stressed rats, or being exposed to previously identified alarm pheromone molecules^2^ increased rats’ freezing to a conditioned odor when tested 24 hr later. Fear memory is specific to the conditioned odor cue, and can exist despite the presence of a different context.

Rat pups have also been shown to acquire an odor aversion when a novel odor is paired with their mother’s fear^22^. Thus, the ability of pheromones to serve as a UCS appears early in the life of the rat and continues to function as observed here. Both neonate and mature pheromone fear conditioning depend on the amygdala. Interestingly, in pups this form of learning is mediated by the main olfactory bulb receiving input from the Grueneberg ganglion system, while here the accessory olfactory bulb mediates the critical input. Whether adult fear learning with pheromone signals is as enduring (over weeks) as that observed in pups^22^ remains to be examined.

Freezing here in the classically conditioned rats (O^+^/S^+^) is correlated with enhanced activation of the MOB and BLA during memory recall. The MOB has been shown to be a critical site for odor associations in multiple learning models^25–29^. The BLA is essential for shock-mediated conditioning including contextual fear conditioning^13,16,30^ and odor conditioning^22^. Hebbian plasticity requires coincident inputs of both CS and UCS onto common postsynaptic cells^31^. While the BLA receives olfactory inputs and somatosensory inputs such as those induced by electrical shock^32,33^, whether MOB neurons receive direct somatosensory inputs is unknown. Alternatively, locus coeruleus (LC) neurons release NE following shock^34,35^. The MOB^36^ and BLA^37,38^ receive extensive LC projections. Odor and NE inputs could converge on both MOB and BLA neurons to initiate plasticity mechanisms. It has been shown that both Hebbian plasticity and neuromodulation by NE are required for tone-shock fear conditioning in the amygdala^39^. MOB plasticity could reinforce BLA potentiation either through its direct projections to the BLA, or *via* the PC or the MeA projections^40–42^, as suggested by our retrograde tracing data. In fact, both the PC and BLA exhibit potentiated odor responses following fear learning in another study^43^. In associative odor reward learning, PC neurons exhibit more reliable activation to the conditioned odor, although the absolute number of activated cells does not increase^44,45^. This may occur during aversive odor shock conditioning as well.

It is intriguing that pheromone-conditioned learning in companion rats leads to enhanced AOB activation to the conditioned odor upon memory recall. Although initially regarded as functionally independent systems^46,47^, with the MOB system being responsible for volatile odorant detection and the AOB system detecting pheromones, recent evidence has revealed that the two systems have considerable overlap in terms of chemosignal detection and the behavioural effects they mediate^48,49^. A subset of vomeronasal neurons express odorant receptors and project to the AOB^50^. The AOB system can thus detect both odorants and pheromones^48,49,51^. We suggest potentiation of AOB neurons is linked to pheromone stimulation acting as a UCS in the associative learning of the CS odor with both signals converging on and potentiating common neurons in the AOB. Another possibility is that amygdala cortifugal input shaped the potentiated responses in the AOB despite an absence of greater activation in the back-projecting cortical amygdala nucleus^52^. The ability of terpinine exposure following a 10 min exposure to the O^+^/S^+^ rat to promote equally strong specific cue odor learning in the companion rat is somewhat surprising, given the long-standing evidence that CS must precede or be contiguous with the US in associative learning. Companion rats were able to associate the pheromone released from a stressed rat with a subsequent odor cue in the absence of the stressed rats, however an association is not formed without the subsequent odor cue (Fig. 1). It is possible that LC NE release during pheromone-induced stress^7^ could prime pheromone-activated neurons for later association with odor activation. The odor stimulus would then subsequently become sufficient to drive AOB neurons in the absence of the pheromone stimulus. Our data showing how β-adrenoceptor blockade prevents pheromone learning is consistent with the involvement of NE. However, our data do not exclude the possibility that other priming effects may occur during social interaction between the stressed and companion rats such as residual odor smell on the stressed rats or ultrasonic communication.

Similar to classically conditioned fear, stressed cage mate-conditioned fear activates the BLA during memory recall. Abolishing plasticity in the BLA by NMDA receptor blockade prevents both classical and pheromone-conditioned fear memory formation. From the BLA, information flows to the medial portion of the CeA which sends output to the periaqueductal grey and hypothalamus to mediate freezing and autonomic responses associated with fear^16^. Elevated CeA activation was observed in both types of learning in our study. Our results regarding the classical and pheromone conditioning pathways are summarized in Fig. 4C.

Functionally speaking, it is advantageous for an animal to associate environmental cues with a potential danger even when he navigates away from the source of the alarm pheromone, as the source of the alarm pheromone, such as a stressed or injured rat, may have parted from the actual site of the danger. This is mimicked in our behavioral paradigm when the companion rats received the pheromone in the home cage from shock-treated rats that had already parted from the shock chamber.

In summary, we now report that alarm pheromone released from a stressed rat can serve as a UCS and produce associative learning in a receiver rat. Whether additional input from the stressed rat primes or amplifies these effects remains to be clarified, but the pheromone alone experiments demonstrate that this input suffices to produce conditioning. Unlike classical odor conditioning that leaves a memory trace in the MOB, pheromone conditioning potentiates AOB activation and the AOB appears to mediate the specific odor cue association as well. However, the two forms of learning activate common fear pathways in the amygdala. BLA plasticity is critical for both classical and pheromone conditioned learning. Our study sheds light on how animals communicate with each other in nature and how they may avoid danger through pheromone-mediated associative learning.

## Electronic supplementary material

Supplementary Figures

## Data Availability

The datasets generated during and/or analyzed during the current study are available from the corresponding author on reasonable request.
