# Supplementary material for: Pheromone-Induced Odor Associative Fear Learning in Rats
Source: Sci Rep. 2018 Dec 7;8:17701. doi: 10.1038/s41598-018-36023-w (PMC6286391; doi:10.1038/s41598-018-36023-w)
Supplement: Supplementary file 1 — Supplementary Figures [file 41598_2018_36023_MOESM1_ESM.pdf]

Supplementary Information

Title: Pheromone-Induced Odor Associative Fear Learning in Rats

Samantha J. Carew[1], Bandhan Mukherjee[1], Iain T.K. MacIntyre[1], Abhinaba Ghosh[1], Sa Li[2], Gilbert J. Kirouac[2], Carolyn W. Harley[3], Qi Yuan[1*]

Supplementary Figure 1

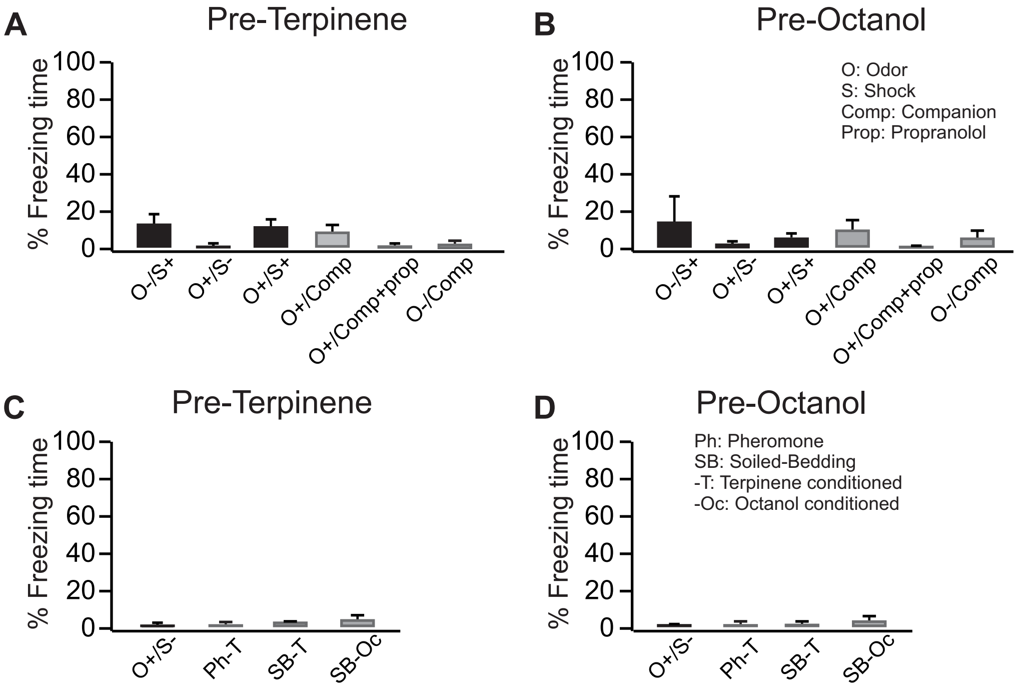

Pre-odor baseline freezing

A-B: Percentage freezing before the odor exposure in Experiment 1 (Figure 1). C-D: Percentage freezing before the odor exposure in Experiment 2 (Figure 3).

Supplementary Figure 2

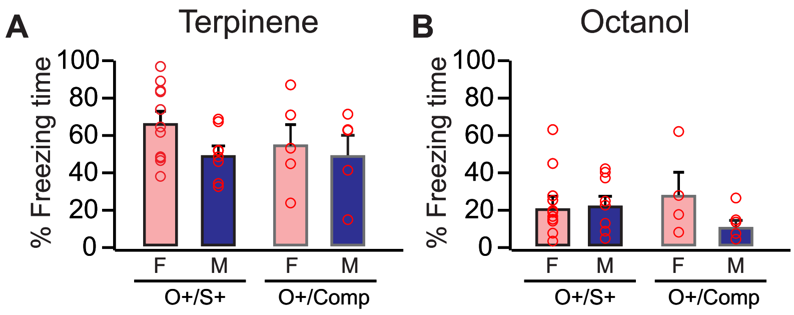

No sex differences in classical and pheromone associative learning

A. Percentage freezing to terpinene in female and male rats in the O^+/S^+ and O^+/Comp groups. B. Percentage freezing to Octanol in female and male rats in the O^+/S^+ and O^+/Comp groups. F, female; M, Male.

Supplementary Figure 3

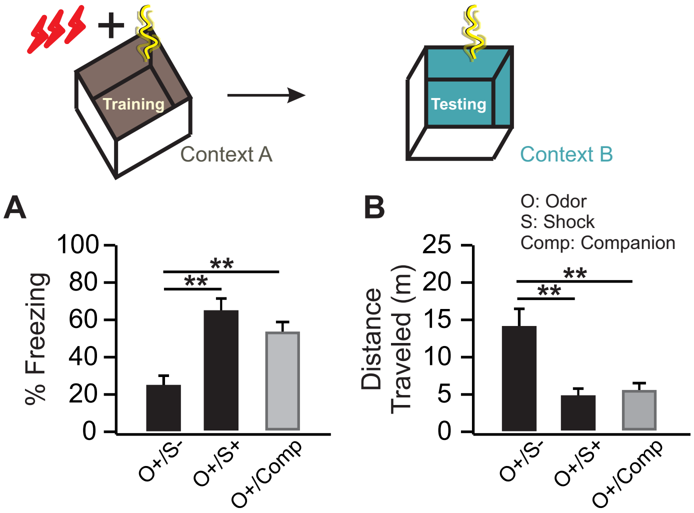

Odor-conditioned rats show fear response to the conditioned odor regardless of context

Rats were trained in a shock chamber (context A) as in Figure 1, and tested in a different context.

A. Percentage freezing to the conditioned odor terpinene. B. Distance traveled in the context B.

Supplementary Figure 4

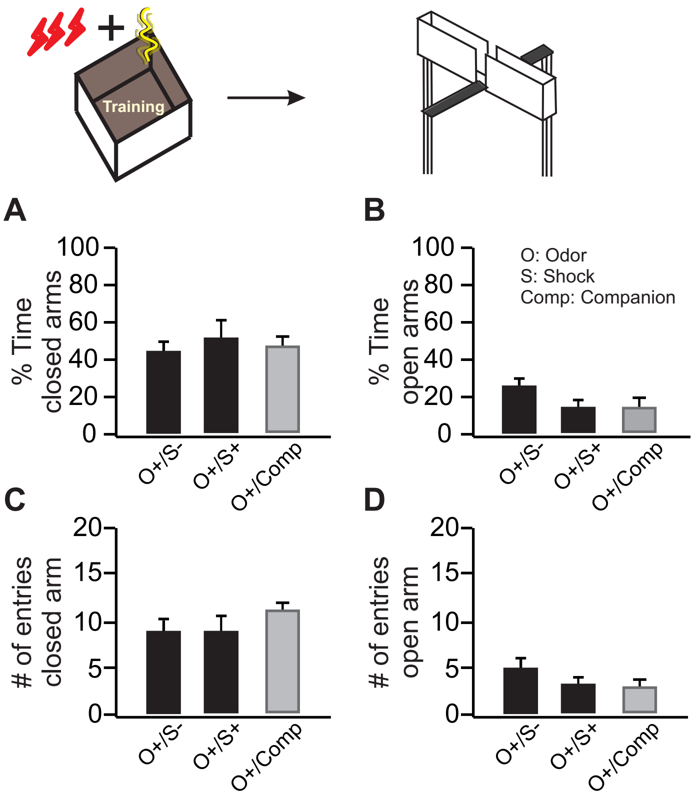

No elevated general anxiety following classical and pheromone associative learning

Anxiety was tested with a wooden elevated plus maze comprised of four 50 cm long and 10 cm wide sections (two open and two closed) merging in a 10 cm square piece at the center. Closed arms are surrounded by 40 cm wall. The maze was placed 50 cm above the floor and a video camera was fixed above the maze to record movements for analysis. Rats were positioned in a closed arm and allowed to roam freely for 5 min. The number of arm entries and time spent in the open vs. closed arms were calculated. If light stimulated rats spent more time in the closed arms than the controls, it would be an indication of anxiety.

A. Percentage time spent in the closed arms. B. Percentage time spent in the open arms. C. # of entries in the closed arms. D. # of entries in the open arms.

Supplementary Figure 5

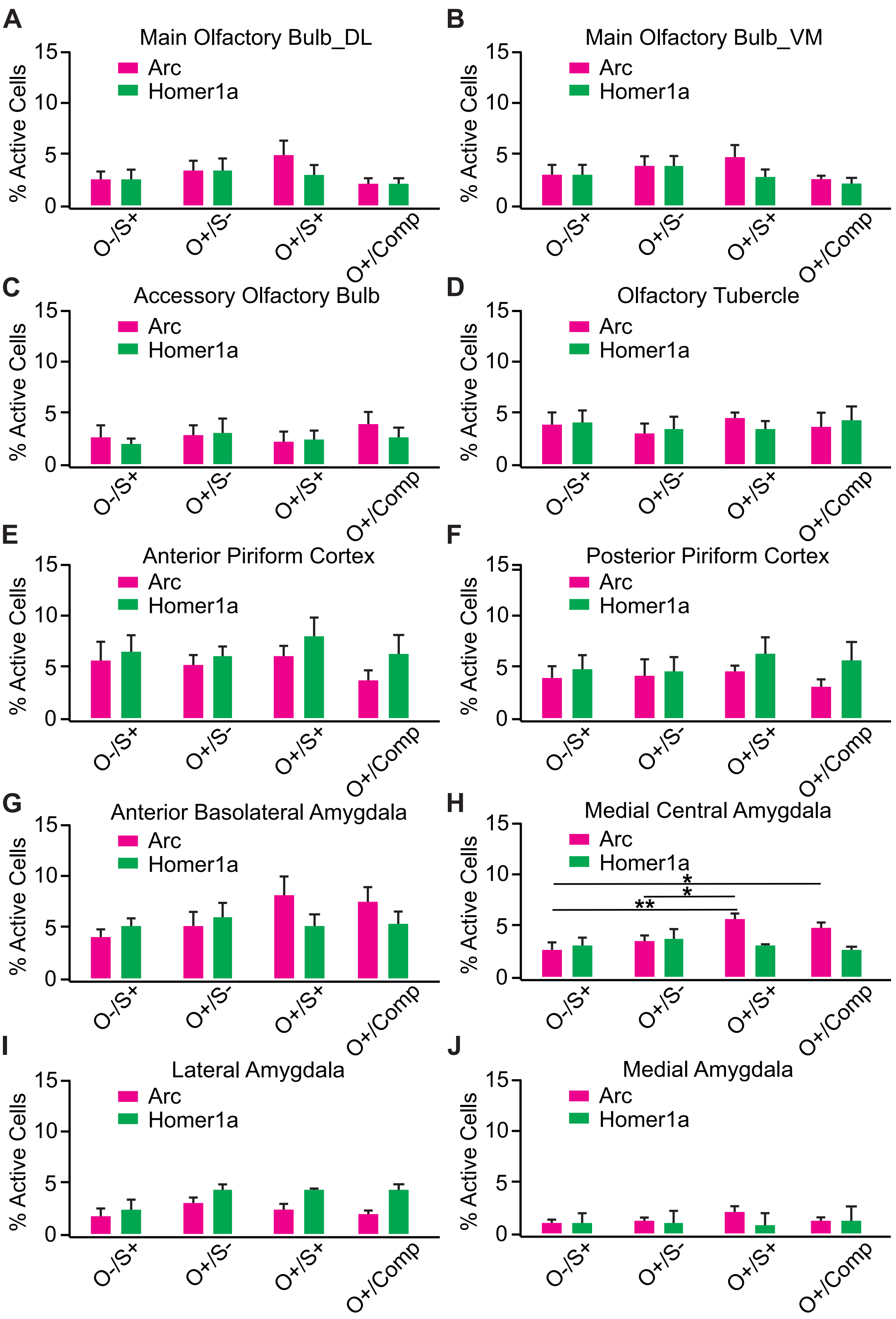

***Arc* and *H1A* activations in various olfactory and amygdala regions**

Arc$^+$ cells are the cells activated by the conditioned odor terpinene. *H1A*$^+$ cells are the cells activated by the control odor octanol. O$^-$/S$^+$, shock only rats; O$^+$/S$^-$, odor only rats that were caged alone; O$^+$/Comp, odor only rats that were caged with odor/shock conditioned rats; O$^+$/S$^+$, odor/shock conditioned rats. *$p<0.05$.

Supplementary Figure 6

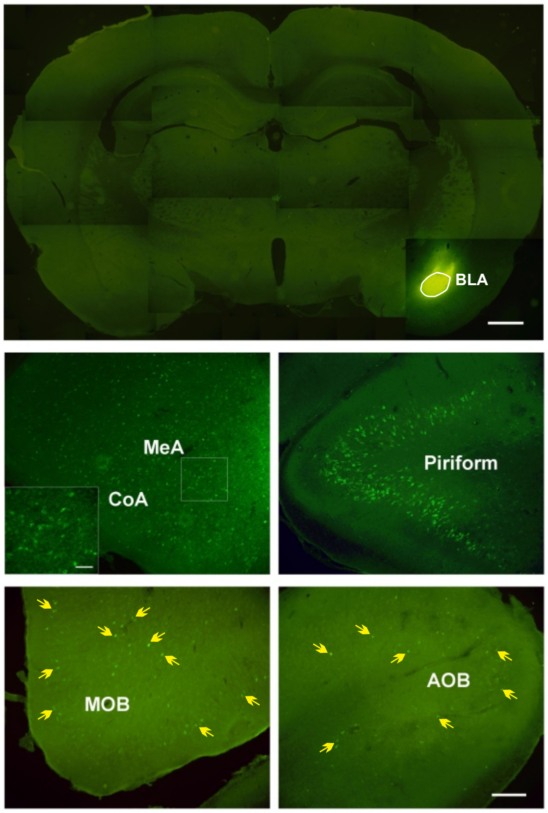

Retrograde tracing from unilateral BLA of a rat

Animals (n=2) were anesthetized with 2-3% isoflurane and given meloxicam (2 mg/kg, s.c.) for post-surgery pain management. Pressure injections of AF-CTb-488 (Invitrogen, USA) were made in the BLA (2.5 mm posterior, 4.9 mm lateral, 7.8 mm ventral relative to bregma and the dural surface of the brain) using glass pipettes with outer diameter of approximately 37 to 40 µm as done previously (Dong et al. 2017). After a 7- to 9-day postoperative survival, animals were deeply anesthetized with 10% chloral hydrate (600 mg/kg, i.p.) and transcardially perfused with 150 ml heparinized saline followed by 400–500 ml ice-cold 4% paraformaldehyde in 0.1 M PB (pH 7.4). The brains were removed and post-fixed in the same fixative for 4–5 h, and cryoprotected in 20% sucrose with 10% glycerin over 2 days at 4 °C. Coronal sections of the brain and olfactory bulb were taken at 50 µm and sections were mounted on slides at every 200 µm for subsequent examination under a fluorescent microscope. Brain sections were examined and photographed using an Olympus BX51 microscope equipped with a digital camera (Spot Insight, Diagnostic Instruments Inc, Sterling Heights, MI, USA) and the images were transferred to Adobe Photoshop CS4 to optimize light and contrast levels.

MOB: main olfactory bulb; AOB: accessory olfactory bulb; BLA: basolateral amygdala; CeA: central amygdala; PAG: periaqueductal grey. Arrows indicate example labelled cells in the MOB and AOB.

Dong, X., Li, S. & Kirouac, G. J. Collateralization of projections from the paraventricular nucleus of the thalamus to the nucleus accumbens, bed nucleus of the stria terminalis, and central nucleus of the amygdala. *Brain structure & function* **222**, 3927-3943, doi:10.1007/s00429-017-1445-8 (2017).
